# A New Treatment for Local Adiposity with Ascorbic Acid and Ascorbyl-Palmitate Solution: Clinical and Histological Study

**DOI:** 10.1007/s00266-020-01865-1

**Published:** 2020-08-14

**Authors:** Antonio Scarano, Andrea Sbarbati, Roberto Amore, Eugenio Luigi Iorio, Giuseppe Ferraro, Domenico Amuso

**Affiliations:** 1 University of Palermo, Master of Techniques of Aesthetic Medicine and Wellness, Palermo, Italy; 2 Department of Neurosciences, Biomedicine and Movement Sciences, Anatomy and Histology Section, School of Medicine, University of Verona, Verona, Italy; 3 Master course in Aesthetic Medicine, Department of Medical, Oral and Biotechnological Sciences, University of Chieti-Pescara, Chieti, Pescara, Italy; 4 Department of Plastic, Reconstructive and Aesthetic Surgery, Università degli Studi della Campania Luigi Vanvitelli, Naples, Italy; 5 Department of Medical, Oral and Biotechnological Sciences, University of Chieti-Pescara, Via dei Vestini 31, 66100 Chieti, Italy

**Keywords:** Local adiposity, Body contouring, Lipolysis injection, Ascorbic acid, Ascorbyl-palmitate

## Abstract

**Background:**

Localized adiposity (AL) is the accumulation of subcutaneous adipose tissue, placed in definite anatomic areas, building up an alteration of the body silhouette. The aim of the present clinical and histological study is to assess the effectiveness of an injectable solution containing sodium salt of ascorbic acid 0.24% and surfactant agent at 0.020% ascorbyl-palmitate (SAP) for treating local adiposity.

**Methods:**

Eighty healthy female adult patients were selected, suffering from local adiposity in the abdominal region. The patients underwent a cycle of 6 sessions, with biweekly treatments, without the addition of any active ingredient. Direct infiltration of pharmacologically active SAP solutions into the adipose tissue with a long needle, very similar to the needles used for spinal anesthesia, was performed. This procedure is quick and painless (does not require any anesthesia) with moderate infiltration speed.

**Results:**

All the patients treated showed good results with good satisfaction of the circumferential reductions. Before treatment: Waist (cm) 78.8 ± 10.6 and hip 93.6 ± 9.0 with WHR 0.84 ± 0.07. After treatment: Waist (cm) 70.8 ± 9.6 and hip 92.6 ± 8.0 with WHR 0.76 ± 0.06. Indeed, signs of adipocyte apoptosis were observed in subcutaneous skin after injection of SAP.

**Conclusion:**

The results showed in the present study suggest that the SAP utilized induces apoptosis of adipocytes and could be of use as a safe and effective method with which to eliminate subcutaneous abdominal fat.

**Level of Evidence IV:**

This journal requires that authors assign a level of evidence to each article. For a full description of these Evidence-Based Medicine ratings, please refer to the Table of Contents or the online Instructions to Authors www.springer.com/00266.

## Introduction

Increased caloric intake and reduction of energy demand can be cause excessive body weight and excessive localized fat. Excessive localized fat and body weight may weaken health, acknowledging different multiple factors such as physical, psychological, and genetic causes. The increasing demand for methods of body fat reduction is maybe driven by the fact that 13% of the world’s adult population (15% of women and 11% of men) were obese in 2016 according to the WHO World Health Statistics Report 2020 [1]

For classifying overweight in adults, we use weight-for-height that is commonly known as BMI. It is calculated by weight in kilograms divided by the square of height in meters (kg/m^2^). The BMI, however, is insufficient for evaluating risk factors and does not take fat distribution into account, but it is associated with health impairment [2]. For this reason, the waist-to-hip ratio (WHR), waist circumference divided by the hip circumference, was proposed as this index takes into account the differences in body structure, fat distribution and has more sensitivity in the prediction of several diseases that can cause mortalities [3, 4]. Furthermore, it is increasingly evident that excess abdominal fat is closely associated with the development of metabolic disturbances [5–7] while accumulation of fat in the lower body is associated with a protective glucose and lipid profile after adjustment for total body fat mass [8, 9].

On the contrary, localized adiposity (AL) is the accumulation of subcutaneous adipose tissue, placed in definite anatomic areas, building up an alteration of the body silhouette. Usually, undesirable fat is situated in the abdomen, flanks, and thighs back, arms, and chest. Excessive localized fat is also an aesthetical problem, physical appearance has great value, and many patients who suffer from excessive localized fat feel weakened with a big impact on relationships with friends or loved ones, the social environment, lowering self-esteem, and affects personal and professional life in general.

Body fat distribution is influenced by genetic makeup [10], epigenetic mechanisms [11, 12], sex hormones, use of glucocorticoids [13]. The reduction in the calories consumed, a balanced diet and lifestyle changes must be recommended for weight loss and the reduction in risk factors.

Liposuction or injection lipolysis techniques have been proposed for removing the excess subcutaneous fat for improving the aesthetical appearance. Liposuction is a gold-standard procedure used in clinical practice [14]; however, it is an expensive and is a surgical procedure which often needs general anesthesia. For these reasons, most studies propose particularly non-invasive techniques for the reduction of subcutaneous fat layers such as injections of lipolysis [15], cryolipolysis [16], radiofrequency ablation [17], and HIFU (high-intensity focused ultrasound) [18]. Today, more patients demand noninvasive techniques for reducing local fat, representing now the fastest growing area of aesthetic medicine. The first technique proposed was infra-adipose injections of phosphatidylcholine and deoxycholic acid which were available for efficacious chemical lipolysis [15]. Vitamin C (ascorbic acid) was used mixed with Klein solution because it increased lipolysis and could improve skin retraction where liposuction did not achieve good results [19]. Many clinical studies report that BMI and fat distribution plasma are inversely related to plasma concentration of AA [20, 21].

In this paper, we aimed to illustrate the efficacy of sodium ascorbate for the treatment of local adiposity. The aim of the present clinical and histological study is to determine the efficacy of an injectable solution containing sodium salt of ascorbic acid 0.24% and surfactant agent at 0.020% ascorbyl-palmitate (SAP) for treating local adiposity.

## Materials and Methods

The study was based in a private multi-specialty medical clinical practice in Montesilvano (Italy), in full accordance with ethical principles, including the World Medical Association Declaration of Helsinki (https://www.wma.net/wp-content/uploads/2018/07/DoH-Oct2008.pdf) and the additional requirements of Italian law. The sample included 80 healthy adult female patients, suffering from local adiposity in the abdominal region. The patients were treated between January 2008 and December 2019. The selected patients were between 25 and 65 years of age, Body Mass Index (BMI) between 23 and 26 with normal weight or slightly overweight without history of adverse health conditions. Each patient signed informed consent on the adopted procedure, and all of the patients knew which pathology the treatment was aimed at. Inclusion criteria for the study included patients with excess adiposity in the abdominal region and who wanted a nonsurgical option for reduction of the same. Exclusion criteria included pregnancy and lactation, menstrual cycle, local infections or skin problems, and anticoagulant medications.

The adipocytolytic agent used was a sodium salt of ascorbic acid 0.24% and surfactant agent at 0.020% ascorbyl-palmitate (SAP) an isotonic solution for sodium chloride in phosphate buffer (Skin-Fat, Ital Farmacia, Rome, Italy). The SAP was introduced via a 5 mL syringe with a 24G needle and a length of 16 cm, very similar to the needles used for spinal anesthesia.

Maintaining the patient seated, infiltration was performed after first disinfecting the area to be treated.

**Fig. 1:**
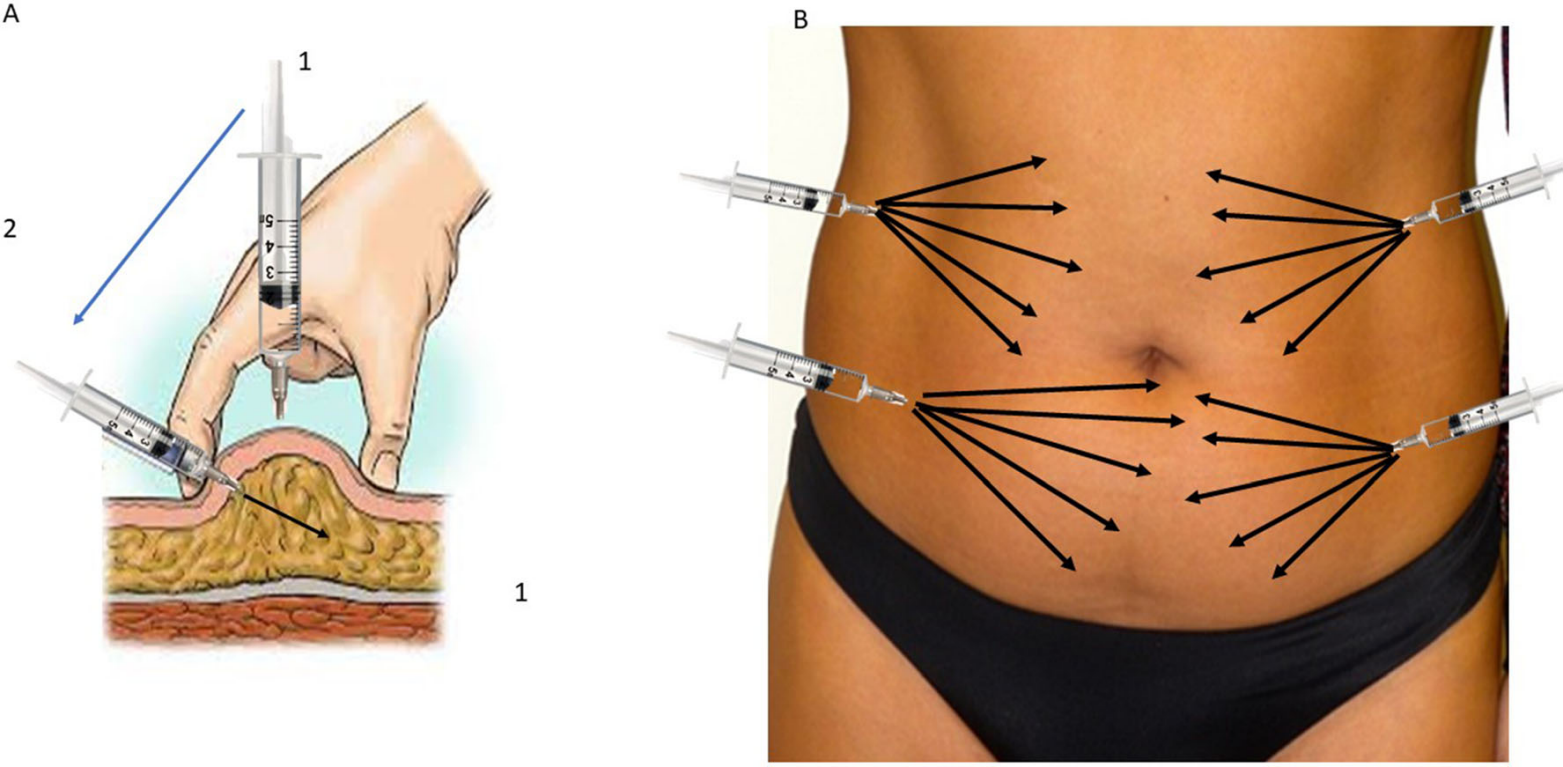
**a** Piercing the skin with and needle and passing through the dermis. After entering for 2–3 cm, it orientated parallel to the skin plane when the depth of the injection was 3–4 cm. **b** The solution was released into the adipose tissue with fan technique

The index finger and thumb of the free hand was used to lift the skin and to exert light pressure on the abdominal area while the needle entered directly on the point of maximum projection of the fat pad to be treated, perpendicular to the skin. After piercing the skin with the needle and passing through the dermis, there was an immediate sensation of loss of resistance to the advancement of the needle. After entering for 2–3 cm, it orientated parallel to the skin plane when the depth of the injection was 3–4 cm (Fig. 1a). This was the signal of having reached the fat under the skin and the right plane, continuing until all 16 cm of the needle entered and the pharmacologically active solution was released into the adipose tissue. During this phase, the needle was neither visible nor palpable on the skin surface, each infiltration of 0.5 mL was a retrograde and fanning insertion (Fig. 1b). The product should not be diluted and the infiltration speed must be moderate. An amount of 0.5 mL of SAP solution was released and while the needle was being removed, the remaining solution (4.5 mL) was gradually released. This procedure was repeated 4 times and a total 20 mL SAP solution was infiltrated into the abdominal region. During all 6 section, a total 120 mL of solution was patient get. Direct infiltration of pharmacologically active solutions into the adipose tissue, with a moderate infiltration speed, is a quick and painless procedure (does not require any anesthesia). While performing the least number of entry points into the skin, it is important to provide a homogeneous coverage of the anatomical region to be treated.

Immediately after the treatment, the use of an icepack was compulsory for 15′ for reducing edema and avoiding pain in the post-treatment phase. The patients underwent a cycle of 6 sessions, with biweekly treatments, without the addition of any active ingredient.

Aesthetic outcomes were evaluated by the authors using preoperative and postoperative photographic documentation and thorough clinical examination and metric documentation with waist-to-hip Ratio (WHR) index. Waist and hip circumferences were measured with the subject in a standing position using a flexible metric measuring tape to the nearest 0.5 cm.

### Histology Processing

We explained to our patients that these biopsies were only for research purposes and did not have an impact on their treatment plan and a second informed consent was achieved. Each patient underwent biopsies at different times during the therapeutic procedure: before the therapeutic procedure and after the last two-week treatment, that is, 14 weeks from the start of the therapy. In total, 160 biopsies were retrieved, 80 before treatment and 80 after treatment. The biopsies were performed with a circular punch biopsy of 2 millimeters diameter (KAI Industries, Oyana, Japan). These are devices with a very sharp stainless steel blade and a plastic handle, each curette is marked with size for easier identification, individually packaged in a sterile sachet. The abdominal area previously treated with SAP was selected and was disinfected with 10% povidone-iodine solution (Betadine, Meda Pharma spa, Italy). This area was stretched with the thumb and index finger of the free hand. The biopsy instrument was pushed in vertically over the skin and rotated downward using a twirling motion created by the dominant hand. Once the punch biopsy entered the dermis into the subcutaneous fat, there was a swinging movement to cut the tissue to the base and then it was removed. After the biopsy, a small hole remained which was covered with a plaster. The wound was closed with a plaster and the cylindrical tissue specimens were stored immediately in 10% buffered formalin and processed for histological analysis. These slides were stained with hematoxylin and eosin observed in normal transmitted light under a Nikon microscope ECLIPSE (Nikon, Tokyo, Japan). Four fields of 2000 μm in diameter to 4000 μm in length were evaluated for each sample.

The biopsies were carried out to document:

- adipocyte volume
- adipocyte number
- inflammatory cells
- vessel number

### Statistical Analysis

A power analysis was performed using clinical software for determining the number of samples needed to achieve statistical significance for quantitative analyses of cell numbers for:

- adipocyte volume
- adipocyte number
- inflammatory cells
- vessel number

A calculation model was adopted for dichotomous variables (yes/no effect) by using the incidence effect designed to discern the reasons (80% for the Test group and 20% for the control group), with alpha = 0.05 and power = 95%.

The optimal number of samples for analysis was 60 patients per group.

Numerical results are presented as the ± SD means of all the experiments.

The data outcome was collected and statistically evaluated by the software package Graphpad 6 (Prism, San Diego CA–USA). The normal distribution of the study data was evaluated by the Kolmogorov-Smirnov test to evaluate the normal distribution. The Mann-Whitney test was performed to compare the study variables means in each group. The level of significance was set at *p *< 0.05.

## Results

**Fig. 2:**
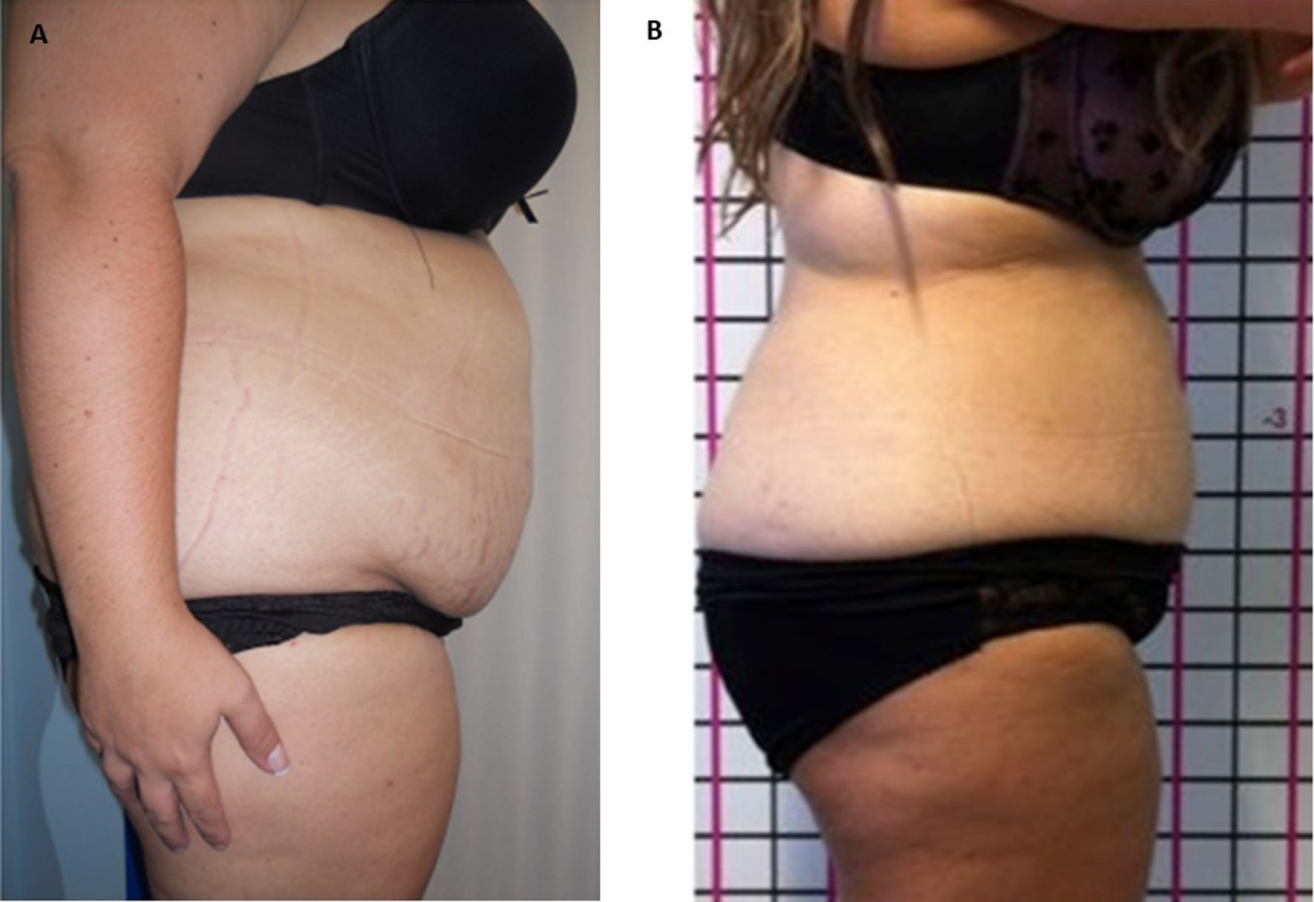
**a** Before treatment **b** After treatment. The abdominal circumferential reductions were achieved

**Fig. 3:**
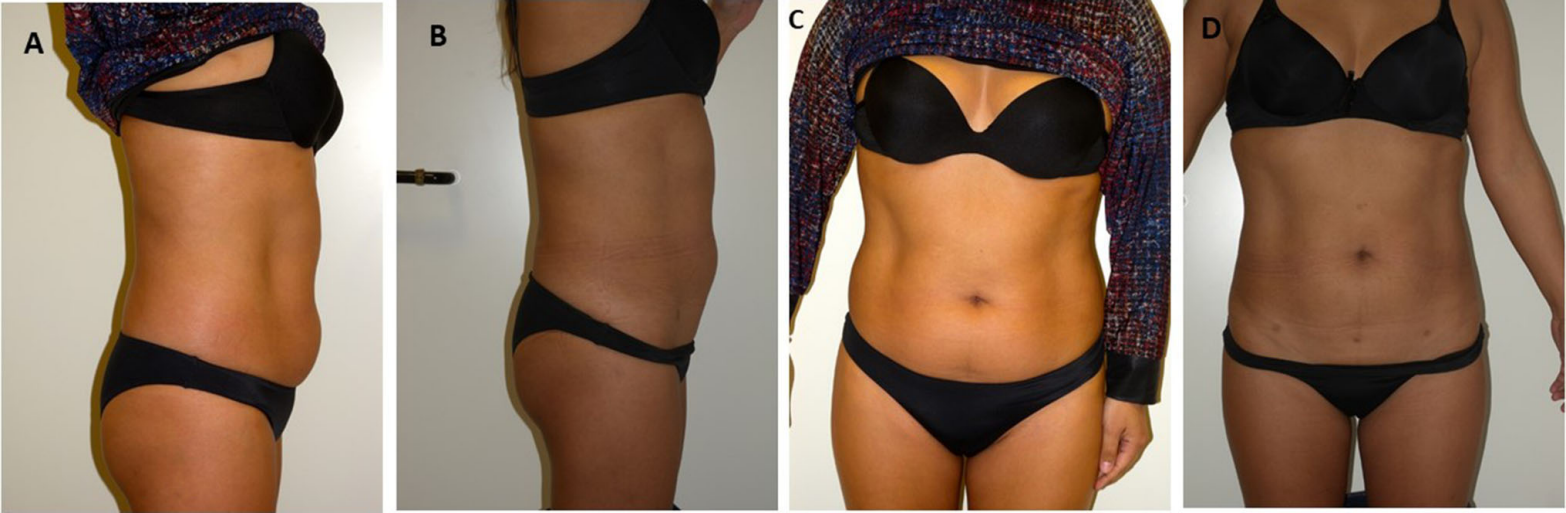
**a**–**c** Before treatment **b**–**d** After treatment. The reduction in the abdominal circumferential is evident

Localized swellings were reported in the abdominal region, no patients reported erythema following the injections, only six patients reported formation of subcutaneous nodules resolved over 7–14 days. All patients treated showed good results with good satisfaction of the circumferential reductions (Figs. 2a, b, 3a–c). Before treatment: Waist [cm] 78.8 ± 10.6 and hip 93.6 ± 9.0 with WHR 0.84 ± 0.07. After treatment: Waist [cm] 70.8 ± 9.6 and hip 92.6 ± 8.0 with WHR 0.76 ± 0.06 (Figs. 2, 3).

The average circumferential reduction after treatment was 8.1(± 9.6) cm while the WHR was decreased to 0.08 (Table 1).

**Table 1:** Summary of the waist, hip, and WHR measurements before and after the treatment

Groups	Waist		Hip		Waist-hip ratio	
	Before	After	Before	After	Before	After
Average (SD)	78.8 ± 10.6	70.8 ± 9.6	93.6 ± 9.0	92.6 ± 8.0	0.84 ± 0.07	0.76 ± 0.06
*p* value	*p *< 0.01		*p *< 0.01		*p *< 0.01	

### Histological Analysis

#### Before Treatment

**Fig. 4:**
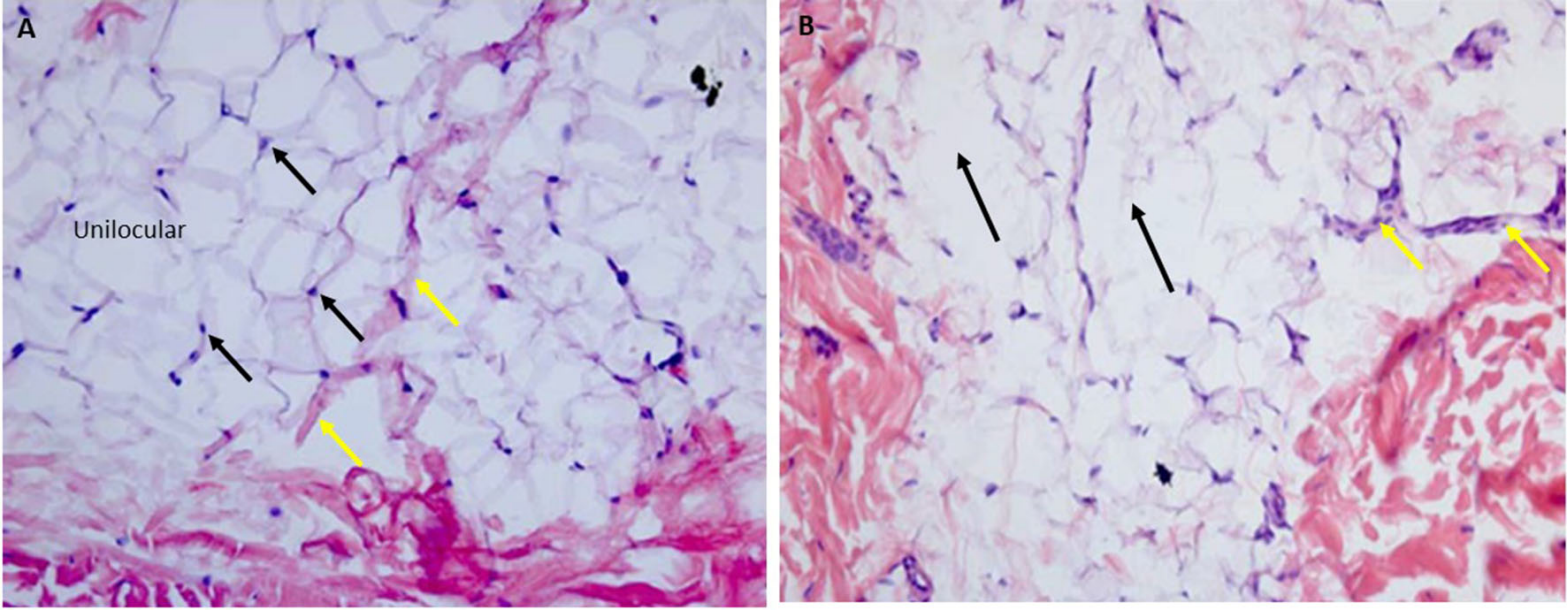
**a** Before treatment. Histological photograph shows mature unilocular, rounded in shape, adipocytes (black arrows), and connective tissue (yellow arrows). **b** After treatment. Shows a typical fat necrosis and macrophages will invade later and take care of the fat debris, and probably leave a certain fibrosis. The adipocytes are irregular in shape with (black arrows), from a probable reaction to an insult or a reparative effect. A few cell nuclei appear absent and other are pale and with vacuoles. The same adipocytes have frosted glass appearance. A few vessels were present (yellow arrows). No infiltration of inflammatory cells was observed. Hematoxylin and eosin X50

A rich vascularization was observed in the adipose tissue, with newly formed small blood vessels accumulating around the adipocytes. Unilocular adipocytes, or white adipocytes rounded in shape and size up to 100–220 µm in diameter, separated by connective tissue septa were present. Many shrunken adipocytes were visible in fat layers with the absence of inflammatory cell infiltration. Additionally, no alteration of the microcirculation was evident before treatment. No apoptotic nuclei showed nuclear fragmentation between adipocytes (Fig. 4a). Few vessels were observed. The histomorphometric results are shown in Table 2.

**Table 2:** Histological means count of Adipocyte Volume, Adipocyte Nuclei, Inflammatory cells infiltrate, and Vessels

Groups	Adipocyte volume		Adipocyte nuclei		Inflammatory cells		Number vessel	
	Before	After	Before	After	Before	After	Before	After
Average (SD)	180 ± 1.4	50.01 ± 0.4	100.5 ± 2.22	30 ± 8.77	2.25 ± 0.7	3.52 ± 0.33	1 ± 4.4	2.5 ± 1.5
*p* value	*p *< 0.01		*p *< 0.01		*p *< 0.01		*p *< 0.01	

#### After Treatment

Unilocular adipocytes, or white adipocytes irregular in shape and size between 50 and 100 µm, in diameter, were found in the specimens. There was an increase in the number of apoptotic nuclei after the treatment (Fig. 4b).

**Fig. 5:**
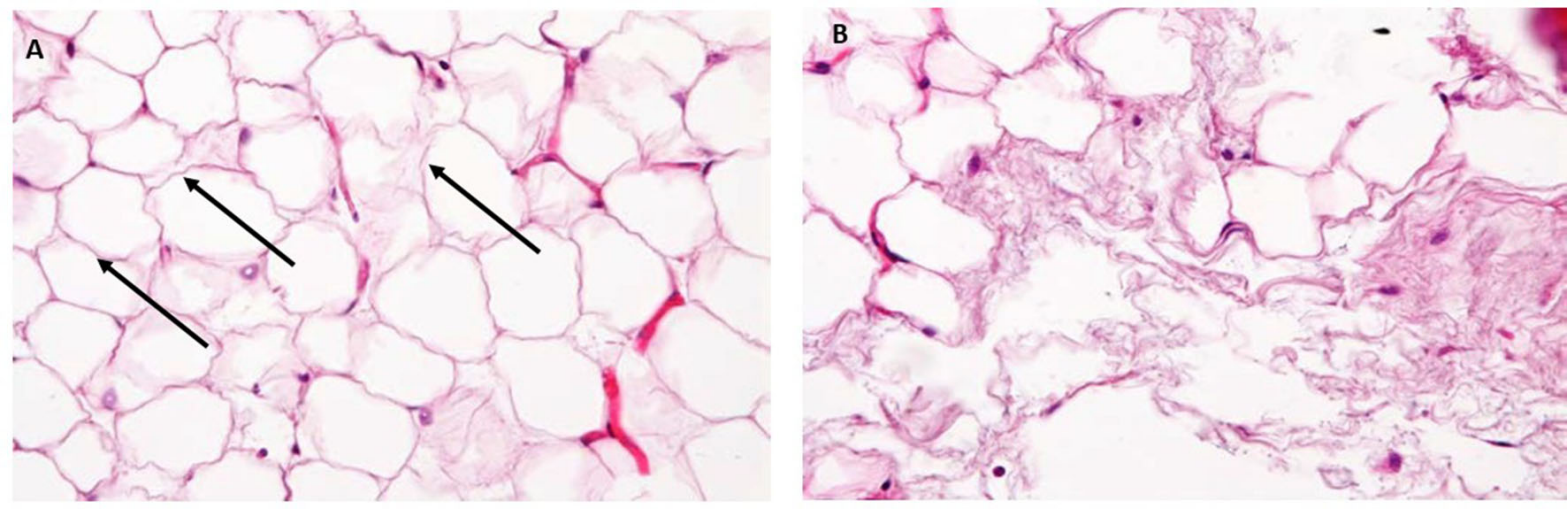
**a** After Treatment. Preview image at high magnification. Histological examination of mature irregular in shape adipocytes and without nuclei (arrows). **b** After treatment. There was an increase in the number of apoptotic nuclei without inflammatory cells

**Fig. 6:**
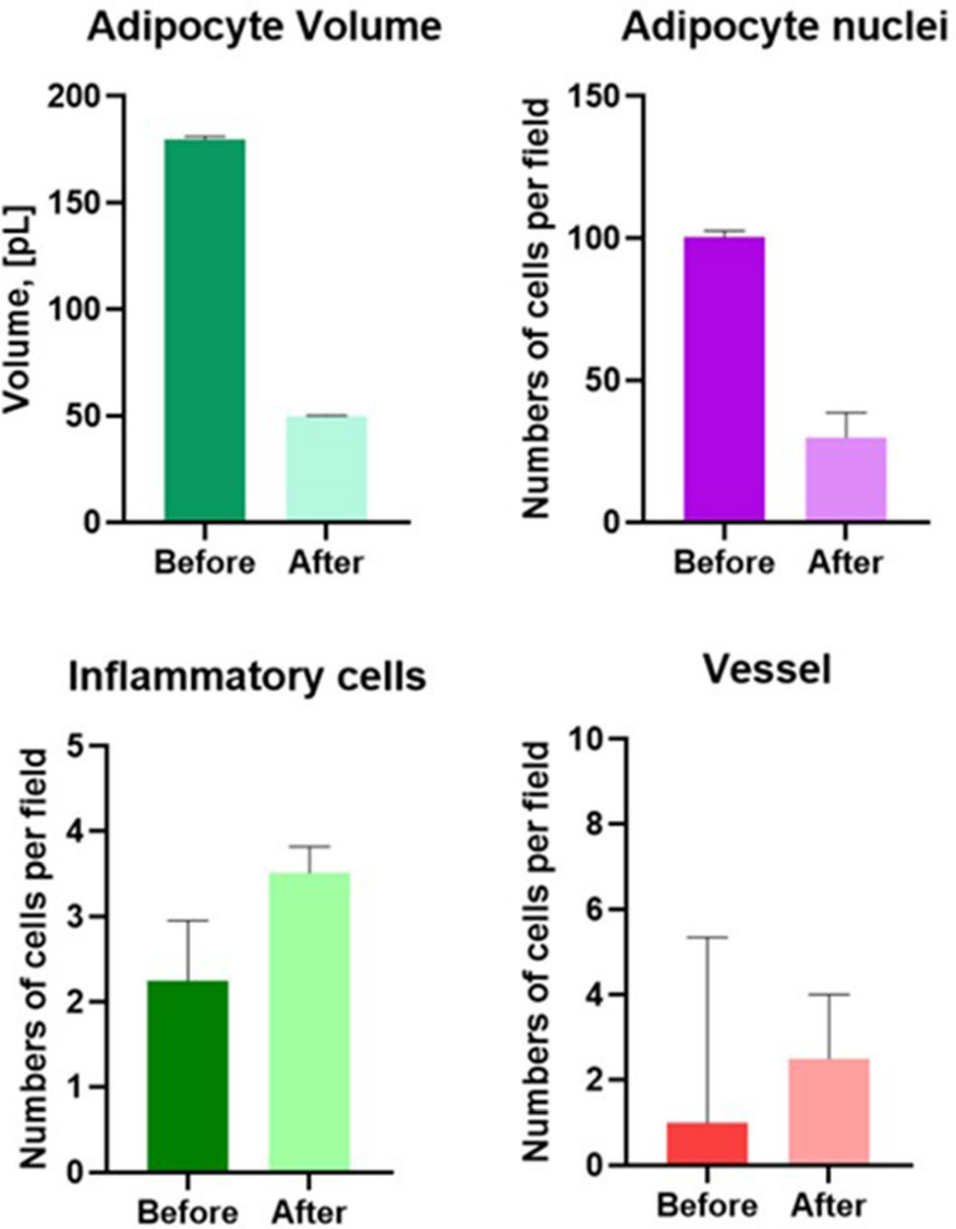
Bar chart of Adipocyte Volume, Adipocyte Nuclei, Inflammatory cells infiltrate, and Vessels. (Mann-Whitney test, *p *< 0.01)

**Fig. 7:**
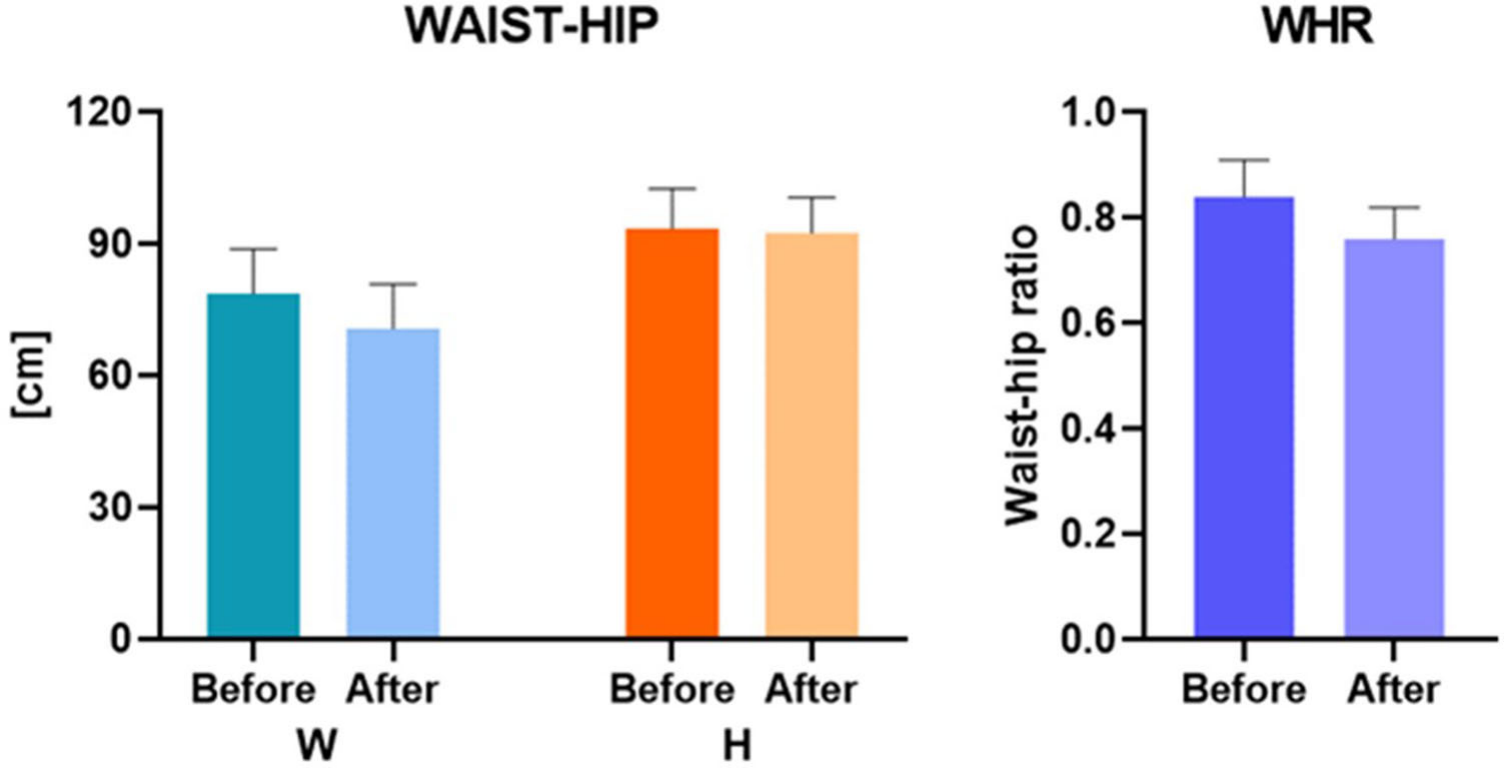
Bar chart of waist, hip clinical measurements, and WHR. (Mann-Whitney test, *p *< 0.01)

The cell nuclei look bigger but pale and with vacuoles inside, from a probable reaction to an insult or a reparative effect; but the nuclei were absent (Fig. 5b). The same adipocytes had “frosted glass” cytoplasm. This aspect is associated with a “rupture” of the adipocytes on granular background (Figs. 4b, 5b). Morphometric analysis of adipocytes indicated that the population of small-size adipocytes was increased and the population of large-sized adipocytes in WAT was decreased (Figs. 6, 7).

The histomorphometric results are shown in Table 2.

## Discussion

The results of the present study indicate that the adipocytolytic agent with SAP is associated with a lipoclase effect and reduction in vital adipocytes without increase in fibrosis. The clinical results showed a statistical signification reduction in waist circumferences and reduction in WHR index. The average circumferential reduction after treatment was 8.1 ± 0.6 cm, while the WHR was decreased to 0.08 ± 0.6.

Localized adiposity (AL) is accumulations of subcutaneous adipose tissues, placed in definite anatomic areas, building up an alteration of the body silhouette. They are characterized by an increase in volume (hypertrophy) and in the number of the adipocytes (hyperplasia), without histological stromal-parenchymal changes of the cellular structure and without modifications of the hypodermal and dermal microcirculation.

In the AL, dermis and epidermis maintain intact their characteristics, without undergoing alterations. AL is a local increase in healthy adipose tissue, without significant clinical signs of disease: there is no edema, no pain, no skin alteration, differently to what occurs with cellulite.

Even histologically, AL do not show pathological changes: adipose tissue is alternatingly hypertrophic and hyperplastic, without anisopoikilocytosis, and is normally vascularized and made up of very large adipocytes, which are greater than 100 μm in diameter and globular, surrounded by a network of reticular fibers, laying one on another with low interposition of intercellular substance.

The reduction in localized fat, intralipotherapy, is used with success in aesthetical medicine, the purpose is creating adipocytolytes with substances by direct injection into fatty tissue with a long needle. This technique, used frequently for over 15 years, uses phosphatidylcholine mixed with sodium deoxycholate as a surfactant agent, a new study while several studies have been carried out where the adipocytolytic agent is sodium deoxycholate and not phosphatidylcholine [22].

In the present study, we chose the adipocytolytic sodium ascorbate mixed with ascorbyl-palmitate as a surfactant agent because the lower vitamin C plasma level is significantly associated with obesity [23]. The increase in levels of vitamin C plasma through supplementation has direct effects on behavioral activity and on adipocyte lipolysis in rats model [24] and its intake promotes weight loss in obese men and women [25, 26]. Animal studies show that diet supplementation of vitamin C reduces by 46% mesenteric adipose tissue mass, adipocyte size and can be used in preventing or treating visceral obesity and glucose intolerance [27]. The ascorbic acid inhibits adipocyte differentiation by inhibiting adenylate cyclase, acting as a global regulator of intracellular cyclic adenosine monophosphate (cAMP) levels and sodium-dependent vitamin C transporter 2 (SVCT2), and is probably involved in these differentiation processes [28] with suppression of peroxisome proliferator-activated receptor α and reduce visceral obesity [29]. Ascorbic acid was also used with intravenous infusion against vascular responses to exercise in obese humans [30] or for reducing allergic response of the skin after local treatment [31], so its administration is safe and adverse reaction free. The physiology of adipocyte is not fully known. High dosage of vitamin C induces adipocyte cell death with autophagy through increases in the levels of intracellular Ca2 + and reactive oxygen species (ROS) and decreases the production of intracellular ATP [32, 33] with the same mechanism induces cancerous cell death [32, 34]. Ascorbyl Palmitate was associated because it presents a lipophilic component with a hexadecanoic chain that facilitates the interaction between the lipid phase and the aqueous phase, increasing the dispersibility and the emulsifying capability [35]. Vitamin C, modulates the release of catabolic inflammatory cytokines, and can regulate the inflammatory process and stimulate the tissue repair [36, 37]. The effect of autophagy may be linked to apoptosis, but not to necrosis or cytoprotective processes. Tissue necrosis and apoptosis are the two major mechanisms of cell death. Apoptosis is a method of programmed cell death to remove cells damaged by noxious agents or disease [38, 39].

Our outcome suggests that SAP increased adipocyte apoptosis. Intra-dipose injection of SAP produces an extensive fat contraction resulting in apoptosis of adipocyte, no adverse events were observed to skin and surrounding tissue. The subjects were normal weight or slightly overweight without complications of any diagnosis of degenerative diseases and lifestyle habits that would have favored aging such as: smoking, alcohol abuse, etc. The interesting study demonstrates that adipocyte apoptosis is an effective and irreversible method for removing subcutaneous fat, while weight loss through diet reduction decreased the volume of adipocytes without decreases in actual fat cell numbers [40, 41]. Therefore, a decrease in the number of adipocytes can be a rational approach to relieve the undesired expansion of adipose tissue. This is an important aspect because the adipocytes have a mean lifespan of 10 years with only 10% of all adipocytes undergo a yearly renewal process [41]. The limitations of the present study are that all subjects were females, and we did not investigate the difference between the two sexes and we have not randomized the patients.

## In conclusions

The treatment with SAP is painless, except for the possible sensation of pain at the site of injection, although the treated patients did not report any sensation of pain. The treatment, once carried out by maintaining sterility requirements and medical common sense, is absolutely safe, and it is effective for reducing undesirable subcutaneous abdominal fat deposits in subjects with normal weight or slightly overweight without disease complications.
